# Crash‐and‐dash: a new era in tree genome editing

**DOI:** 10.1111/nph.70118

**Published:** 2025-04-01

**Authors:** Chung‐Jui Tsai

**Affiliations:** ^1^ Warnell School of Forestry and Natural Resources University of Georgia Athens GA 30602 USA; ^2^ Department of Genetics University of Georgia Athens GA 30602 USA; ^3^ Department of Plant Biology University of Georgia Athens GA 30602 USA

**Keywords:** base editing, CRISPR, perennial, *Populus*, transgene‐free editing

## Abstract

This article is a Commentary on Hoengenaert *et al*. (2025), **247**: 224–232.

In less than a decade since the first demonstrations of CRISPR genome editing of agronomic genes in several tree species (Zhou *et al*., 2015; Jia *et al*., 2016; Ren *et al*., 2016), this disruptive technology has been deployed for a growing number of traits in both basic and applied research. The precision and efficiency of CRISPR editing allow for the recovery of null mutants in the first generation (Zhou *et al*., 2015; Elorriaga *et al*., 2018; Muhr *et al*., 2018), which is a significant benefit for perennial trees with long generation times. The stability of editing outcomes over multiple years or clonal propagation cycles (Bewg *et al*., 2022; Chen *et al*., 2023; Goralogia *et al*., 2024) is another key advantage since most woody perennials are vegetatively propagated in commercial operations. However, in many countries, gene‐edited trees with stably integrated T‐DNA face the same regulatory hurdles as traditional transgenics, slowing field trial characterization and the integration of transgenesis with conventional breeding (Boerjan & Strauss, 2024). In this issue of *New Phytologist*, Hoengenaert *et al*. (2025; pp. 224–232) demonstrate transgene‐free editing in poplar (*Populus tremula* × *alba*) that shows promise for wide adoption. The CRISPR‐edited, transgene‐free canker‐resistant citrus (*Citrus sinensis*) trees (Su *et al*., 2023) have recently been approved by USDA‐APHIS and are exempt from regulation by the US Environmental Protection Agency (EPA) for commercial production. The work by Hoengenaert *et al*. suggests a similar path could be followed for purpose‐grown plantations for bioenergy, bioproducts, and biomaterials.

‘*Long‐read sequencing of two such events revealed no traces of T‐DNA or the binary vector backbone, confirming that they were derived from transient transformation*…’

Earlier attempts to produce transgene‐free mutants of perennial crops primarily relied on direct delivery of CRISPR reagents into protoplasts; however, this approach was only successful in taxa with robust protoplast regeneration systems (Su *et al*., 2023). A more widely applicable method using *Agrobacterium*‐mediated transformation for transient expression of CRISPR components without antibiotic selection successfully recovered mutants free of T‐DNA (Chen *et al*., 2018). This study reported an efficiency of *c*. 8% based on the visual reporter phytoene desaturase and an elaborate amplicon deep‐sequencing screening approach in the easily transformable tobacco (*Nicotiana tabacum*) model (Chen *et al*., 2018). Further improvements were achieved by targeting the *acetolactate synthase* (*ALS*) gene, either through CRISPR knockout coupled with homology‐directed repair or by base editing, for site‐directed modifications to confer chlorsulfuron herbicide resistance (Danilo *et al*., 2019; Veillet *et al*., 2019). The overall efficiency of edited plants without transgene integration ranged from 5% to 13% in tomato (*Solanum lycopersicum*) and potato (*Solanum tuberosum*) (Danilo *et al*., 2019; Veillet *et al*., 2019). These advancements led to the development of a co‐editing strategy with both positive and negative selection schemes, greatly simplifying the recovery of edited plants for molecular analysis (Huang *et al*., 2023). In this co‐editing strategy, a cytosine base editor is used to target *ALS* and other genes of interest, while green fluorescent protein (GFP) serves as a counter‐selection reporter for the presence or absence of T‐DNA. Recovery of herbicide‐resistant and GFP‐negative plants signifies successful base editing without GFP integration, thereby narrowing the plant population for detailed molecular assessments (Huang *et al*., 2023). This co‐editing strategy successfully generated transgene‐free mutants in tobacco, tomato, potato, and citrus pummelo (*C. maxima*) in the first generation, though with a wide range of efficiency (2–42%) that varied by target site and species (Huang *et al*., 2023).

This co‐editing strategy was also adapted by Hoengenaert *et al*. with a slightly different configuration for lignin biosynthetic pathway manipulation in poplar. Plants were regenerated on chlorsulfuron media conferred by *ALS* mutations with no counter‐selection. As expected with positive selection, base editing was detected at a very high rate for the *ALS* genome duplicates. The co‐editing efficiency was much lower for the *caffeoyl‐CoA O‐methyltransferase1* (*CCoAOMT1*) target, with only one‐third of the shoots regenerated on herbicide media harboring mutations derived from either base editing or indels. Encouragingly, about half of the chlorsulfuron‐resistant shoots were PCR‐negative for two T‐DNA regions adjacent to the right border and left border sequences. Long‐read sequencing of two such events revealed no traces of T‐DNA or the binary vector backbone, confirming that they were derived from transient transformation (Hoengenaert *et al*.).

A current limitation of this promising technology is the low mutation rate at the target (nonselected) site in woody perennials. In citrus, 96% of chlorsulfuron‐resistant shoots were GFP‐negative; however, only four of the 103 GFP‐negative shoots were transgene‐free based on PCR, and only two harbored biallelic or homozygous edits at the target site – the canker susceptibility gene *LOB1* promoter (Huang *et al*., 2023). This resulted in a transgene‐free editing efficiency of 4%, or 2% when only null mutations at the target site were considered (Huang *et al*., 2023). In poplar, only five of 74 chlorsulfuron‐resistant shoots were PCR‐negative for T‐DNA and nonchimeric (*c*. 7%), but no transgene‐free null mutants for *CCoAOMT1* were recovered (Hoengenaert *et al*.), indicating room for improvement.

Potential factors for optimization include CRISPR reagents that directly impact editing efficiencies, such as different Cas nucleases (Cas9 vs Cas12), base editors (Jin *et al*., 2020), and Polymerase III promoters (Deguchi *et al*., 2024). Improving tissue culture organogenesis handling can reduce the occurrence of chimeric shoots (shoots regenerated from a mixture of differentially edited or nonedited cells). Given the low edit rates at the target sites, incorporating negative selection can help enrich for putatively T‐DNA‐free events to facilitate molecular screening, as reported in the citrus study (Huang *et al*., 2023). In this context, Hoengenaert *et al*. conducted preliminary testing of the *Escherichia coli cytosine deaminase* (*codA*) gene as a potential negative selection marker (Osakabe *et al*., 2014) that has also been used in other transient transformation experiments (Bánfalvi *et al*., 2020). Finally, advances in *Agrobacterium* strain modification by manipulating host defense responses (Raman *et al*., 2022; Yang *et al*., 2023) and binary vector engineering via directed evolution (Szarzanowicz *et al*., 2024) can significantly enhance transient transformation efficiencies.

Transgene‐free editing offers numerous advantages beyond regulatory and public acceptance considerations. For outcrossing perennial trees, vegetative propagation is critical to ensure true‐to‐type inheritance of desirable traits. Standard agricultural practices like selfing or backcrossing are not applicable due to self‐incompatibility and long reproductive cycles. Compared with other methods for removing transgenes after integration, which are time‐demanding and rarely scarless, transgene‐free editing is the most practical approach to generating edited trees without foreign DNA in the first generation.

Rigorous field trials are the necessary first step to assess the performance of edited trees under natural environments, across multiple growing seasons, and to perform risk assessments. Unfortunately, stringent regulation of genetically modified organisms has made this critical research component cumbersome and slow. Transgene‐free editing of perennial trees should alleviate some of these regulatory concerns, facilitating field studies for trait evaluations. With the rapidly evolving innovations in the CRISPR field, further improvements in transgene‐free editing efficiency are expected in the coming decade, which will propel bioengineering advances in long‐lived woody perennials.

## Disclaimer

The New Phytologist Foundation remains neutral with regard to jurisdictional claims in maps and in any institutional affiliations.
